# Development and Validation of a Green Analytical Method for Calcium Determination in Pharmaceuticals Using Curcumin: A Sustainable Approach

**DOI:** 10.1155/ianc/6832626

**Published:** 2025-11-21

**Authors:** Nim Bahadur Dangi, Hemraj Sharma, Dipak Paudel, Hari Prasad Sapkota

**Affiliations:** ^1^ Pharmaceutical Sciences Program School of Health and Allied Sciences Pokhara University Pokhara 30 Nepal pu.edu.np; ^2^ Department of Pharmacy Rapti Technical School Rapti Dang Nepal; ^3^ Central Department of Chemistry Tribhuvan University Kirtipur Kathmandu Nepal tribhuvan-university.edu.np; ^4^ Department of Chemistry Prithvi Narayan Campus Tribhuvan University Pokhara Nepal tribhuvan-university.edu.np; ^5^ Department of Pharmacy Shree Medical and Technical College Bharatpur Nepal shreemedical.edu.np

**Keywords:** calcium, curcumin, green analytical chemistry, green reagent, ICH guidelines, Nepal

## Abstract

**Background:**

Reliable determination of calcium in pharmaceuticals is crucial for quality control, yet conventional methods often rely on synthetic chelating agents such as EDTA, which raise environmental and safety concerns. Curcumin, the principal polyphenolic compound in *Curcuma longa* (turmeric), exhibits strong metal‐chelating properties through its β‐diketone structure. This study explores the use of curcumin as a natural and sustainable chelating agent for the spectrophotometric determination of calcium in pharmaceutical formulations.

**Method:**

Curcumin was extracted from turmeric rhizomes collected from different altitudes in Nepal using ethanol and was allowed to form a stable color complex with various calcium‐containing drugs, such as calcium gluconate, calcium lactate, and calcium docusate. The method was optimized by adjusting reagent volume, pH, and buffer volume. Validation was carried out according to ICH guidelines, evaluating linearity, precision, accuracy, and robustness. The environmental impact was assessed using Green Analytical Procedure Index, Analytical Eco‐Scale, and AGREES tools. The Click Analytical Chemistry Index was used to analyze overall sustainability, while the Carbon Footprint Reduction Index was applied to evaluate the prime environmental impact of the already developed analytical laboratory procedures.

**Results:**

The highest curcumin yield was from the Kaski region. The optimized method produced a stable yellow‐orange complex with maximum absorbance at 430 nm. It demonstrated high precision (%RSD < 2%), accuracy (recovery: 101.63% and 102.01%), and robustness with a stable reaction lasting up to 4 h. The environmental assessment confirmed its sustainability and eco‐friendliness, with a very good score reflecting minimal solvent use and no hazardous waste.

**Conclusions:**

This study successfully developed a green, cost‐effective, and reliable method for calcium analysis in pharmaceutical products using curcumin as a natural reagent. Its simplicity and environmental sustainability make it a promising alternative to conventional techniques for calcium analysis.

## 1. Introduction

Calcium is an essential mineral that contributes significantly to various biological functions, such as maintaining healthy bones and teeth, supporting muscle contractions, facilitating nerve signaling, aiding in blood clotting, and forming calcium salts such as gluconate, lactate, and docusate [1]. Calcium deficiency can lead to several health problems, including osteoporosis, rickets, and dental issues. Therefore, accurately determining the calcium content in pharmaceutical products is critical to ensure their safety and efficacy [2]. Conventional analytical methods are commonly based on titration or spectrophotometry, which depends heavily on synthetic chelating agents such as ethylenediaminetetraacetic acid (EDTA). While effective, these reagents have serious drawbacks: they are often nonbiodegradable, require high energy consuming instruments, generate hazardous waste, and may involve toxic solvents or harsh reaction conditions. Such practices contradict the current emphasis on green chemistry and sustainable analytical procedures.

To overcome these challenges, natural reagents are being explored as eco‐friendly substitutes. Plant‐derived compounds not only reduce chemical hazards but also align with the principles of sustainability by utilizing renewable resources. Turmeric (*Curcuma longa*), in particular, offers significant promise. Its principal bioactive compound, curcumin, possesses a β‐diketone moiety that readily chelates divalent and trivalent metal ions and is widely used as a spice in both traditional medicine and cooking, well known for its numerous health benefits, including its anti‐inflammatory and antioxidant effects [3]. The pharmaceutical industry, particularly sectors dedicated to anticancer drug formulations, represents the largest application segment, making up more than 50% of the global market, with the food and cosmetics industries following close behind [4]. The chelation process results in the formation of stable complexes accompanied by a distinct yellow‐orange coloration, which can be easily monitored spectrophotometrically. This unique combination of chelation ability and visible color change makes curcumin an attractive alternative for replacing synthetic reagents in calcium analysis. However, its chelating properties have also been explored in analytical chemistry, particularly in the determination of metal ions [5]. Various green analytical chemistry (GAC) methods have been developed to analyze calcium tablets using UV–visible spectrophotometer [6], colorimeter [7, 8], ion exchange chromatography [9], and thermogravimetry [10]. All the above methods focus on method development and validation for calcium but fail to address the laboratory hazards associated with their analysis. Hence, it was considered necessary to develop an eco‐friendly analytical method to address this issue, which can be covered by using GAC principles that employ various analytical tools to evaluate the eco‐friendliness of the method [11].

The present study focuses on developing eco‐friendly, reliable, and cost‐effective analytical methods for determining the calcium content in different tablet formulations, which involve simple complex formation and require no toxic reagents or chemicals for analysis. Using the readily available turmeric with very low cost and high safety assurance really helped in conducting and completing the analytical work in an eco‐friendly mindset. The green profile was validated using eco‐scale penalty points, the Green Analytical Procedure Index (GAPI), and the AGREES tools to minimize hazardous exposure to both the analyst and the environment when implementing the proposed methods [12–14]. AGREE sample preparation tool (AGREEprep) was also applied to assist AGREE to ensure the environmental impact of sample preparation by analyzing 10 specific green chemistry parameters [15]. The GAPI assessment was utilized to evaluate and compare the greenness level of the proposed and existing methods. Additionally, the methods’ blueness and whiteness were assessed using the Blue Applicability Grade Index (BAGI) and the RGB 12 model, respectively, for comparative analysis [16, 17]. A comprehensive sustainability comparison was conducted between the proposed and reported methods based on CACI and CaFRI relative scoring, considering the specific parameters of each approach [18, 19].

To date, no research has investigated the application of turmeric powder as a chelating agent for calcium determination in pharmaceutical products. Therefore, this study aims to develop a green analytical approach using turmeric powder for calcium determination. The proposed method is expected to provide accurate, precise, and selective results comparable to conventional methods while offering a sustainable, environmentally friendly alternative to toxic reagents that generate hazardous waste.

## 2. Materials and Methods

### 2.1. Materials and Reagents

A double‐beam UV–visible spectrophotometer (Agilent, Cary 60 UV–Vis) with a 10 mm quartz cuvette was used to record the response as absorbance. HPLC (Agilent 1260 Infinity II) with an Agilent Pursuit C18 column (150 mm × 5.0 mm i.d. × 5 μm) was used for the quantification at 425 nm of the extract (20 μL), with 0.2% acetate buffer and acetonitrile at a ratio of 40:60 as the mobile phase in a gradient elution mode. The flow rate was set to 1.2 mL/min with a column temperature of 25°C. Ethanol, calcium gluconate, calcium lactate, calcium docusate, and calcium carbonate were used for the analysis. The freshly harvested and washed rhizomes of *Curcuma longa* (Figure 1) were collected from three different altitudes in Nepal: Salyan (2600 ft), Kaski (5700 ft), and Ilam (9800 ft) above sea level. The rhizomes were shade‐dried and homogenized into a powder, as shown in the supporting file (Figure S1).

Figure 1Freshly harvested rhizomes (a) and washed rhizomes (b).

**Figure:**
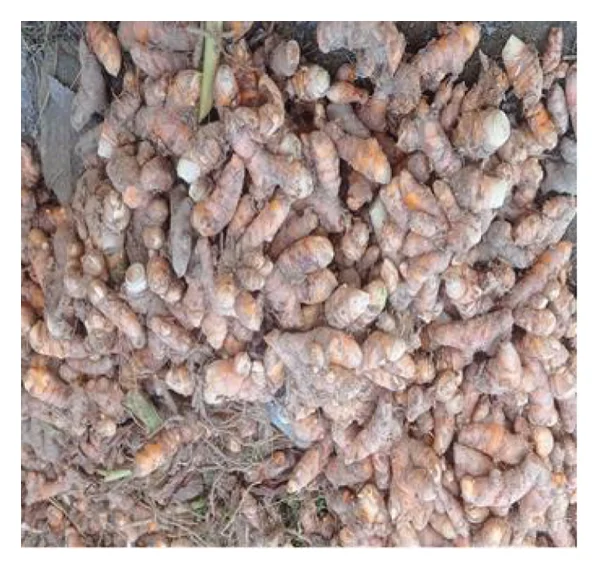
(a)

**Figure:**
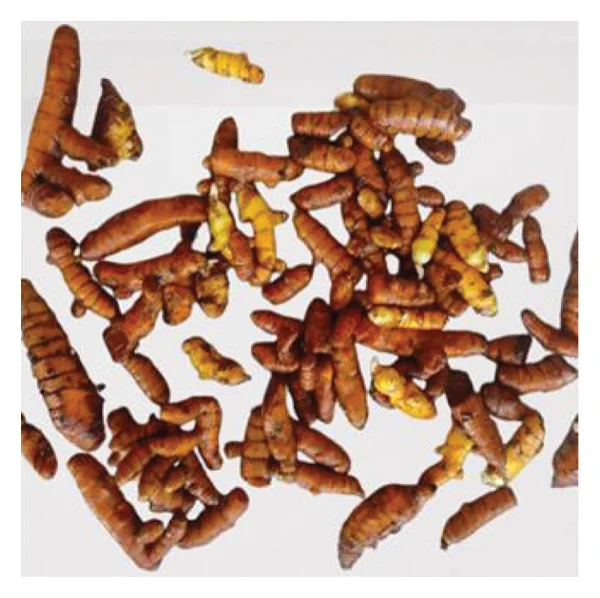
(b)

### 2.2. Research Design and Research Station

The research design was experimental and was conducted in the Pharmaceutical Sciences laboratories at the School of Health and Allied Sciences, Pokhara University. All necessary equipment required for this research was available in the laboratory.

### 2.3. Preparation of Plant Extract

The dried samples were ground in an electric grinder (SKU: YS‐3750U) into a coarse powder. The extraction was carried out using the double maceration method in 70% ethanol for 48 h [20]. The ethanolic extracts were then filtered through Whatman filter paper and dried using a rotary evaporator and a desiccator. The yield percentage was calculated after complete drying. The extract was transferred to a Petri dish, weighed, and allowed to dry completely in the desiccator.

### 2.4. Preparation of Natural Reagent

A total of 100 g of the dried extract was accurately weighed and added to an extraction flask containing 250 mL of deionized water. The mixture was stirred using a magnetic stirrer for 30 min to ensure complete extraction. It was then filtered through filter paper to remove any solid debris. The filtered solution was diluted to a final volume of 100 mL with deionized water to obtain the desired concentration. The preparation of the dried powder of *Curcuma longa* and the reagents used in the extraction process followed standard protocols.

### 2.5. Method Development

#### 2.5.1. Selection of Suitable Wavelength, pH, and Other Parameters

To determine the wavelength maxima (*λ_*max) of the color complex, spectrophotometry was performed by gradually increasing the wavelength from 400 nm to 800 nm and recording the absorbance at each wavelength. The wavelength with the highest absorbance was selected as the optimal wavelength for analysis. The pH of the phosphate buffer and the volume of the reagent needed to form a stable color complex were also taken in consideration for method development.

#### 2.5.2. Mechanism of Color Production

**Figure 2:**
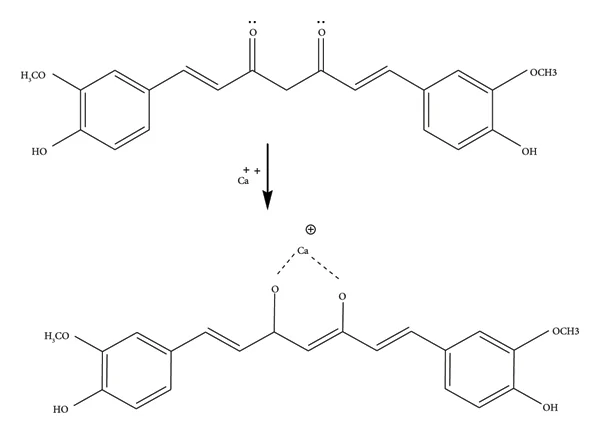
Ca^2+^ reacting with curcumin to form a metal–ligand complex.

Turmeric contains curcumin, an active pigment. Curcumin has nonbonded electrons, which, when it comes in contact with metal ions like Ca^2+^, form a metal–ligand complex, resulting in a color change from its original color (Figure 2). When extracted using ethanol, pure curcumin produces a yellow‐colored solution. When mixed with a calcium‐containing drug (such as calcium gluconate, calcium lactate, and calcium docusate), the color changes from yellow to orange or reddish‐brown in the pH range of 7.5–8.5. The newly formed color complex showed a change in the absorption shift, which was used for the quantification using UV–visible spectrophotometer.

## 3. Results

### 3.1. Extraction of Natural Reagent From Turmeric

**Figure 3:**
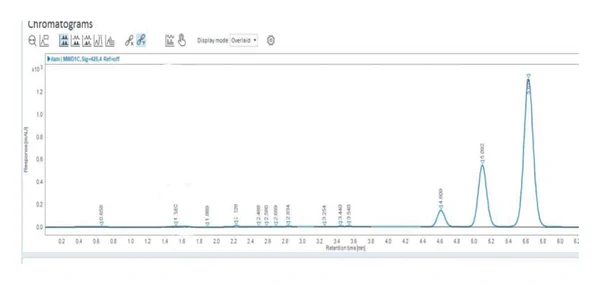
HPLC chromatogram of turmeric extracts showing curcumin.

The HPLC chromatogram was developed to authenticate curcumin, as shown in Figure 3. The curcumin peak in the sample was confirmed by injecting a standard curcumin under identical conditions. In the reference chromatogram, the retention time (*t*_*R*) of curcumin was 5.6; therefore, the peak near 5.6 in the sample was identified as curcumin. The chromatogram of the standard is shown in supporting file (Figure S2).

### 3.2. Optimization of Method Parameters

**Figure 4:**
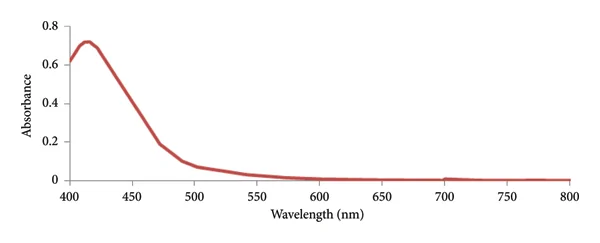
Absorption maxima of color complex.

The curcumin obtained from the rhizomes of *Curcuma longa* was used as a natural reagent and was allowed to react with calcium salt in the presence of a phosphate buffer to form a yellowish‐orange product with an absorption maximum of 430 nm as shown in Figure 4. To achieve the formation of the color complex, various parameters like reagent volume, pH, buffer volume, and color complex stability play a crucial role; hence, their effect was studied in terms of optimization. The optimization was initially achieved by adjusting the reagent volume (1–4 mL), selecting the appropriate pH, determining the required volume of phosphate buffer, and assessing the stability of the complex.

### 3.3. Optimization of Reagent Volume, Reaction Stability, and pH

The optimization process began by adjusting the reagent volume (1–4 mL), with maximum absorbance observed at 2 mL. Consequently, 2 mL was selected as the optimal reagent volume, as detailed in Table 1. The optimal reaction time for the reaction between the drug and curcumin was 1 min, and the color complex remained stable for 4 h (Table 1). After 4 h, some of the complexes settled at the bottom of the volumetric flask, resulting in slight precipitation, which may have contributed to the decrease in absorbance values. Phosphate buffers with pH values ranging from 5.8 to 7.5 were prepared, and absorbance was measured. The highest absorbance was observed at pH 5.8, which was selected as the optimal pH for the analysis, as shown in Table 1.

**Table 1:** Optimization of method parameters.

**Optimization of natural reagent (*λ* = 430 nm)**	
**Volume (mL)**	**Absorbance**

1	0.1803
2	0.3965
3	0.3289
4	0.3943

**Optimization of Reaction Stability**	
**Time (h)**	**Absorbance**

1	0.725
2	0.792
3	0.736
4	0.742
5	0.618
6	0.601

**Optimization of pH**	
**Buffer pH**	**Absorbance**

5.8	0.393
6.5	0.103
7.5	0.216

**Optimization of Volume of Buffer**	
**Volume of Buffer (mL)**	**Absorbance**

1	0.241
2	0.408
3	0.349
4	0.272

### 3.4. Validation

The analytical method was validated to confirm that its performance characteristics align with the requirements of the intended analytical use. The validation was carried out in accordance with ICH Q2 (R1) and Q2 (R2) Guidelines [21, 22]. Various validation parameters including linearity, accuracy, precision, specificity, and robustness were evaluated as summarized in Table 2.

**Table 2:** Validation parameters for the determination of curcumin by the proposed spectrophotometric method.

**Parameter**	**Values**

Range (μg·mL^−1^)	1.0–6.0

*Linearity*	
Slope	0.101
Intercept	0.423
Correlation	0.999
Accuracy^a^ (Mean ± SD)	101.83 ± 0.190

*Precision (RSD%)*	
Intraday precision^b^	1.14–1.25
Interday precision^c^	1.31–2.06
Robustness^d^ (% RSD)	0.955

^a^The accuracy at 4 μg·mL^−1^ was assessed at three concentration levels: 80%, 100%, and 120% for calcium carbonate.

^b^The intraday precision was determined as the average of three concentrations (2, 3, and 4 μg·mL^−1^), each repeated three times within the same day.

^c^The interday precision was determined as the average of three concentrations (2, 3, and 4 μg·mL^−1^), each repeated three times over three consecutive days.

^d^Robustness: %RSD of three replicates (*n* = 3) analyzed after applying deliberate changes.

Linearity was confirmed within the concentration range of 1–6 μg·mL^−1^, demonstrating a strong correlation with a coefficient (*R* ^2^) of 0.999, satisfying Beer’s law of absorptivity. Precision studies were established as intraday (analyzed three times within the same day) and interday (evaluated over three consecutive days), using concentrations of 2, 3, and 4 μg·mL^−1^ within the linearity range. The % RSD value for the intraday precision was found to be below 2%, and the interday precision was up to 2.06%. The percentage recovery method was used to assess the accuracy of 4 μg·mL^−1^ by using three levels of concentrations, i.e., 80%, 100%, and 120%, and it ranged from 101.63% to 102.01%, as shown in Table 2. The limit of detection (LOD) and limit of quantification (LOQ) for the procedure were determined using the standard deviation of the absorbance values and the slope of the calibration curve and were found to be 0.449 μg·mL^−1^ and 1.36 μg·mL^−1^, respectively. The robustness of the method was established by altering the *λ*_max of the color complex (*λ*_max 430 nm) by ±1.0 nm. The % RSD was found to be 0.944, demonstrating the method’s stability and reliability under small deliberate changes.

### 3.5. Application of the Developed Method

The assay of calcium‐containing drugs, such as calcium gluconate injection, calcium lactate tablets, and calcium docusate capsules, was conducted to establish the application of the developed method. The procedure was carried out in triplicate, and the outcomes are presented in Table 3, which includes the percentage of drugs present in the formulation, as well as the mean and standard deviation.

**Table 3:** Assay of calcium‐containing drugs.

**Formulation**	**Labeled claim**	**Amount found (mean ± SD)**	**Assay %**

Calcium Gluconate Injection	100 mg·mL^−1^	3.897 ± 0.02	97.44
Calcium Lactate Tablets	500 mg	4.029 ± 0.026	100.74
Calcium Docusate Capsule	240 mg	4.06 ± 0.035	101.56

### 3.6. Greenness Evaluation of the Method

Various analytical methods have been developed to date for the analysis of calcium salts, but none have focused on the use of green reagents. Therefore, this method is the first to provide such information. The greenness of the method was assessed using various green analytical tools, including the GAPI, AES, and AGREE. The blueness and whiteness of the method were established by BAGI and RGB12 methods, respectively. The overall sustainability of the method was also established and compared with the reference method using Click Analytical Chemistry Index (CACI) tool. GAPI emphasizes the assessment of hazardous solvents by the National Fire Protection Association (NFPA) guidelines and utilizes a Toxic Release Inventory (TRI) list to identify relevant solvents. Its pictogram incorporates three distinct colors green, yellow, and red reflecting varying toxicity levels. This color scheme corresponds to the toxicity of solvents, with red indicating high toxicity, yellow indicating moderate toxicity, and green indicating low or nontoxicity [12]. The pictogram for GAPI is added in Figure 5(a), satisfied the criteria and confirming the proposed method as eco‐friendly. The detailed GAPI description is added in supporting file (Table S3).

Figure 5Greenness evaluation of the developed method: (a) GAPI, (b) AES, (c) AGREE and AGREEprep, (d) BAGI, (e) RGB12 algorithm, (f) CACI, and (g) CaFRI assessment tools.

**Figure:**
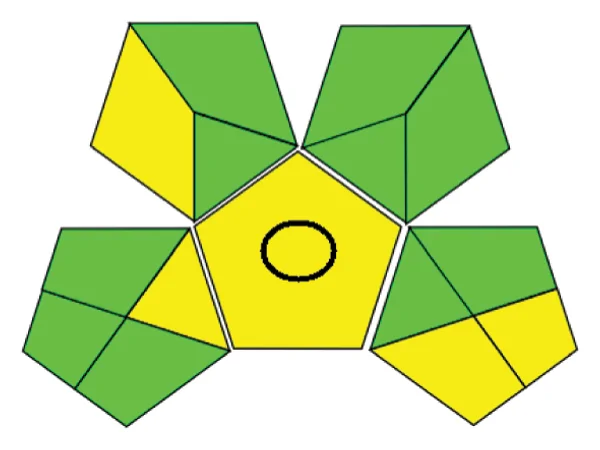
(a)

**Figure:**
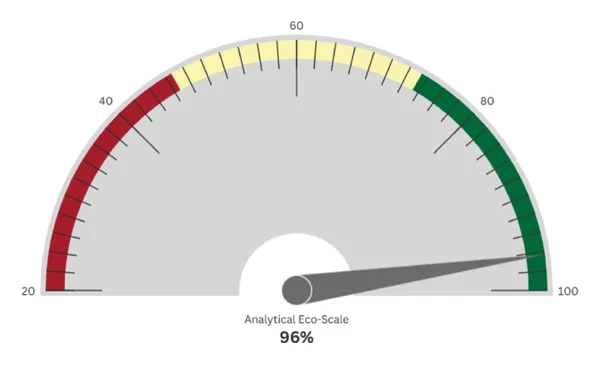
(b)

**Figure:**
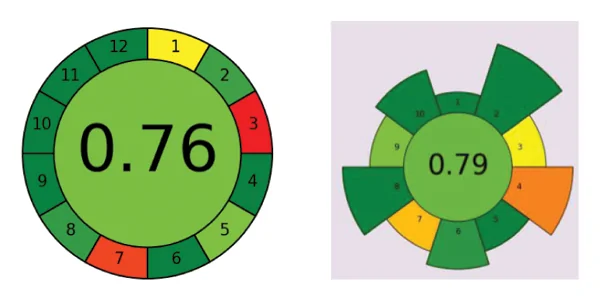
(c)

**Figure:**
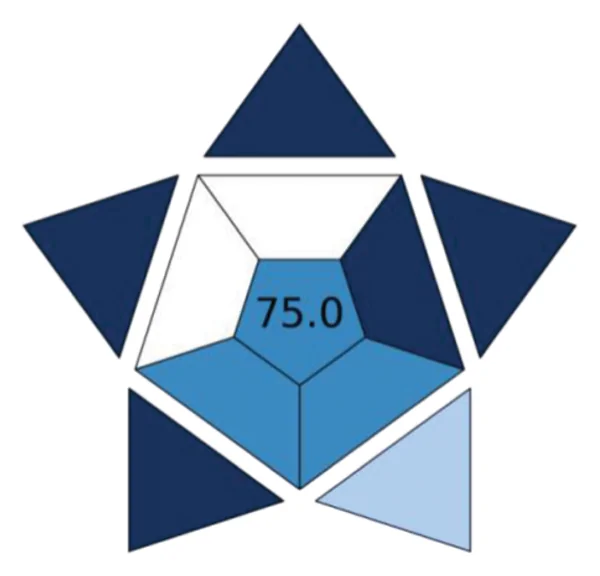
(d)

**Figure:**
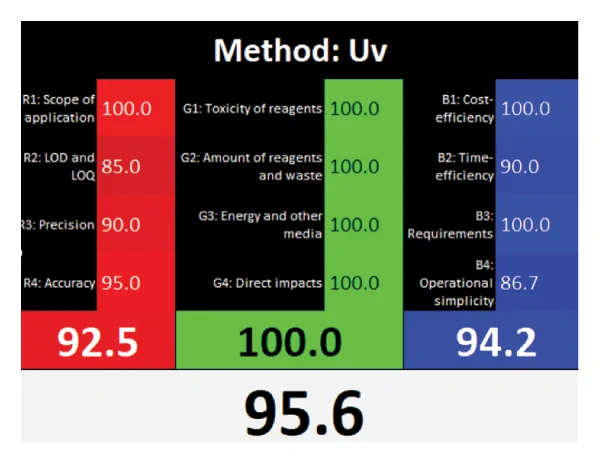
(e)

**Figure:**
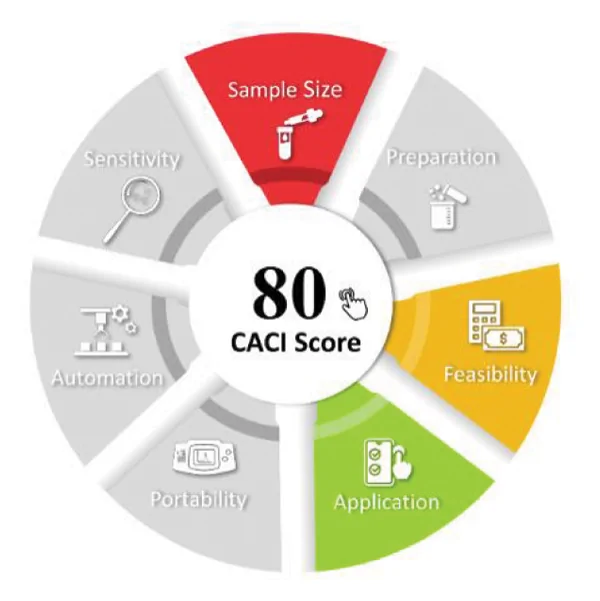
(f)

**Figure:**
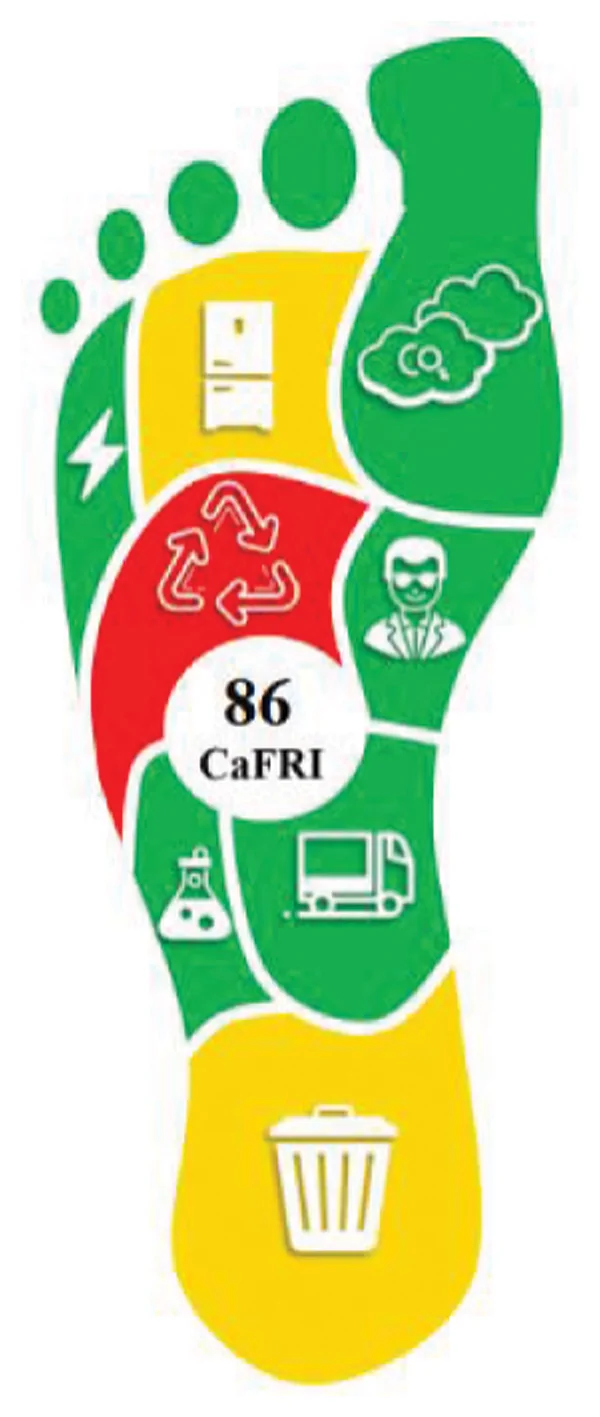
(g)

AES was calculated based on penalty points assigned for various factors such as reagents used, chemicals used, energy consumption, and waste generated. An eco‐scale score of 100 indicates the perfect greenness of the method. A score of more than 75, 50–75, and less than 50 indicates excellent, fair, and deficient green methods, respectively [13]. The eco‐scale score of the introduced method was 96, as detailed in Figure 5(b); the detailed AES description was added in supporting file (Table S4).

AGREE provides a graphical interface that assigns scores from 0 to 1, with 1 reflecting full compliance with green principles [14]. In this study, AGREE yielded a score of 0.76 (Figure 5(c)), highlighting sample preparation and extraction as the most environmentally critical stages. To evaluate these, the AGREEprep tool assesses 10 green chemistry parameters—including sampling, resource use, sustainability, waste, efficiency, automation, energy, handling, and safety—each scored 0–1 and visualized in a color‐coded pictogram [15]. The developed method achieved an AGREEprep score of 0.79 (Figure 5(c)), with mostly green and yellow indicators, confirming its eco‐friendly profile.

BAGI offers an innovative way to assess the practicality and feasibility of analytical methods, focusing on real‐world applications in white analytical chemistry (WAC). The BAGI evaluation process involves 10 steps: type of analysis, single or multielement analysis, analytical method, simultaneous sample preparation, sample preparation, sample throughput, reagent and equipment, preconcentration, degree of automation, and sample quantity [23]. BAGI generates an asteroid pictogram with scores ranging from 25 to 100, where higher values reflect greater practicality and feasibility. The developed method achieved a BAGI score of 75 (Figure 5(d)), demonstrating its sustainability.

The RGB12 algorithm was employed in this study to assess the whiteness of the method, developed by using UV‐spectrophotometry, for the estimation of calcium in various dosage forms [17]. The whiteness score for this UV‐spectrophotometric method was found to be 95.6 (Figure 5(e)), suggesting the method as a good eco‐friendly option.

CACI introduces a groundbreaking approach to chemical analysis, inspired by the simplicity, speed, and reliability of click chemistry. By emphasizing quick, user‐friendly, and practical methods, CACI streamlines procedures, conserves resources, and enhances efficiency especially for on‐site or real‐time analysis. Unlike GAC, which focuses on sustainability, or white analytical chemistry (WAC), which emphasizes inclusivity and balance, CACI offers modular, time‐efficient, and accessible solutions. This approach fills a critical gap by promoting straightforward, reliable techniques, much like glucose meters deliver fast and intuitive results [18]. The ease and applicability of these methods are captured in the CACI chart (Figure 5(f)).

CaFRI is a comprehensive tool for evaluating the environmental sustainability of analytical laboratory procedures, focusing primarily on carbon footprint. It assesses both the method and its laboratory context, considering factors such as energy use, emission sources, mitigation strategies, sample handling, waste management, recycling, and chemical usage [19]. The results are visualized in the CaFRI chart (Figure 5(g)).

## 4. Discussion

This study provides important insights into the development of a green, efficient method for calcium analysis using curcumin extracted from *Curcuma longa*. The UV–visible method utilized a natural reagent (green reagent) to analyze calcium in pharmaceutical formulations. Its capability to quantify the studied drugs in pharmaceutical products makes it a highly effective, green, sensitive, and cost‐efficient spectrophotometric method. The curcumin extraction from rhizomes collected at different altitudes yielded varying concentrations, with the highest yield from Kaski (8.53 g/100 g), and this sample was used for method development. Previous studies reported similar trends where altitude and geographical location influenced the yield and quality of curcumin due to variations in climatic conditions and soil composition [24].

Extraction of turmeric was carried out with water, which led to the formation of a sticky extract from which complete removal of moisture was extremely difficult; also, after a few days, microbial growth occurred. Hence, the solvent was changed from water to ethanol, which is also considered a green solvent with minimal exposure hazards. The use of ethanol as an extraction solvent is consistent with its effectiveness, as reported by [25], who found that ethanol not only facilitates efficient extraction but also aligns with green chemistry principles by reducing the need for hazardous solvents. However, our study reports slightly higher yields compared to some prior studies, which could be attributed to differences in rhizome sourcing or extraction techniques. The ethanol extract was free of moisture and was used for the method development and validation of calcium‐containing drugs.

The chemometric method generally uses a chemical entity to develop a color, whereas in this method, plant extract was used as a reagent to establish the color, which is the novelty of this research. Various green and blue eco‐scale tools also provided documented evidence for the greenness, blueness, and whiteness of this method. The validation of the proposed method was carried out according to ICH guidelines, enabling its application for the determination of the studied drugs in pharmaceutical products.

Patel et al. [26] developed an analytical method for the estimation of calcium in herbo‐mineral formulation (Maxcal‐C Tablet) by atomic absorption spectrophotometry (AAS). This method determined the amount of Ca^2+^ in the tablet by AAS (34.90%) and compared the result by performing complexometric titration against EDTA using Calcon as an indicator (25.12%). However, the percentage purity of the results obtained in the developed method was compared ranging from 97% to 101%, showing that the method is more selective and sensitive for the analysis of calcium. Also, AAS typically requires significant amounts of energy to operate, especially in techniques like flame AAS (which requires continuous fuel and oxidant consumption) or graphite furnace AAS (which requires high temperatures for atomization). This high energy consumption is not aligned with the green chemistry goal of minimizing energy use. Different acids like HCl, HNO_3_, HF, and H_2_O_2_ were utilized for digestion. These reagents can be hazardous, corrosive, and nonenvironmentally friendly, leading to the generation of toxic waste that needs careful disposal. The greenness of the reference method was also established by GAPI, CaFRI, and CACI assessment tools, and the pictogram suggested the method as less eco‐friendly as only a few analytical steps were green. The eco‐friendliness comparison of the reference and developed methods was also established, which showed the developed method to be eco‐friendlier and more sustainable. The detail was incorporated in supporting file (Table S5).

In the titrimetric method using chemical indicators like Calcon, long exposure can cause eye, skin irritation, and respiratory tract. So, the alternative approach has been made by replacing the synthetic chemical, high energy consuming equipment, and increasing waste generation by the simple, cost‐effective, efficient analytical technique with turmeric as a reagent, which is cost‐effective, as it is inexpensive and widely accessible, especially in regions where turmeric is a common household item. This makes the method practical for resource‐limited settings or laboratories looking for low‐cost alternatives to conventional calcium analysis techniques. Curcumin’s presence in turmeric, being a natural, biodegradable, and readily available substance, contributes to an environmentally friendly approach. The simplicity of the method also minimizes energy consumption and waste production, supporting sustainable analytical practices.

A unique aspect of this study is the evaluation of the method’s environmental impact using multiple green chemistry metrics, such as GAPI, Analytical Eco‐Scale, and AGREE. The GAPI analysis highlighted the eco‐friendly nature of the method, with minimal solvent use and no hazardous waste. The method’s eco‐scale score of 96/100 indicates excellent adherence to green principles, superior to the green metrics scores of some established methods, such as those reported by [26], which typically scored in the range of 70–90 for analytical procedures using synthetic reagents. Also, the overall sustainability developed by CACI with a score of 80 ensured the superior sustainability of the developed method.

## 5. Conclusion

This study developed and validated a green analytical method for calcium analysis using curcumin extracted from *Curcuma longa* rhizomes, with the highest yield obtained from Kaski. The method was optimized for reagent volume, pH, and buffer volume, yielding a stable yellowish‐orange complex with maximum absorbance at 430 nm. The procedure demonstrated high precision, accuracy, linearity, and robustness, with % RSD values below 2%, recovery rates exceeding 101%, and stability for up to 4 h. The method was evaluated for its environmental impact using green analytical tools such as GAPI, Analytical Eco‐Scale, and AGREES, confirming its eco‐friendliness with minimal solvent use and no hazardous waste. Moreover, the method’s practicality was supported by a high BAGI score. The novel tools CACI and CaFRI introduced an innovative approach to represent the overall sustainability of the method, with carbon footprint prioritized as the primary environmental impact. So, this study presents a precise, efficient, and sustainable method for calcium analysis, offering a promising green alternative for pharmaceutical and environmental applications.

## Conflicts of Interest

The authors declare no conflicts of interest.

## Funding

This research was supported by Pokhara University Research Center (PURC), Pokhara University, Nepal, through the Faculty Research Grant (Grant no: 10/079/080).

## Supporting Information

A total of five supporting figures and tables (S1–S5) are included as supporting information. Turmeric powder samples collected from different regions of Nepal (Figure S1); the reference chromatogram of curcumin (Figure S2); the Green Analytical Procedure Index (GAPI) (Table S3); the analytical eco‐scale (Table S4); and a comparative evaluation of green analytical tools between the reference and proposed methods (Table S5).

## Supporting information


**Supporting information** Additional supporting information can be found online in the Supporting Information section.

## Data Availability

The datasets generated during or analyzed during the current study are available from the corresponding author on reasonable request.
