# Correction to “Development and Validation of a Green Analytical Method for Calcium Determination in Pharmaceuticals Using Curcumin: A Sustainable Approach”

**DOI:** 10.1155/ianc/9807981

**Published:** 2026-06-15

N.B. Dangi, H. Sharma, D. Paudel, and H.P. Sapkota, “Development and Validation of a Green Analytical Method for Calcium Determination in Pharmaceuticals Using Curcumin: A Sustainable Approach,” *International Journal of Analytical Chemistry*, vol. 2025 (2025), https://doi.org/10.1155/ianc/6832626.

In the article titled “Development and Validation of a Green Analytical Method for Calcium Determination in Pharmaceuticals Using Curcumin: A Sustainable Approach,” Dipak Paudel was missing from the author list.

Dr. Paudel contributed to the HPLC analysis of samples and standards, processed the chromatographic data, and curated the dataset used in the study.

The corrected author list should read as follows:

[“Nim Bahadur Dangi^1,∗^, Hemraj Sharma^2,∗^, Dipak Paudel^3,4^, Hari Prasad Sapkota^5^”

^1^Pharmaceutical Sciences Program, School of Health and Allied Sciences, Pokhara University, Pokhara‐30, Nepal

^2^Department of Pharmacy, Rapti Technical School, Rapti, Dang, Nepal

^3^Central Department of Chemistry, Tribhuvan University, Kirtipur, Kathmandu, Nepal

^4^Department of Chemistry, Prithvi Narayan Campus, Tribhuvan University, Pokhara, Nepal

^5^Department of Pharmacy, Shree Medical and Technical College, Bharatpur, Nepal].

We apologize for this error.

