# Supplementary material for: Development and Validation of a Green Analytical Method for Calcium Determination in Pharmaceuticals Using Curcumin: A Sustainable Approach
Source: Int J Anal Chem. 2025 Nov 21;2025:6832626. doi: 10.1155/ianc/6832626 (PMC12662665; doi:10.1155/ianc/6832626)
Supplement: Supporting information — Additional supporting information can be found online in the Supporting Information section. [file 6832626.f1.docx]

**Supplementary File**

**Development and Validation of a Green Analytical Method for Calcium Determination in Pharmaceuticals Using Curcumin: A Sustainable Approach**

**Nim Bahadur Dangi^1#^, Hemraj Sharma^2*^,** Hari Prasad **Sapkota^3^**

**^1^Pharmaceutical Sciences Program, School of Health and Allied Sciences, Pokhara University**

^2^Department of Pharmacy, Rapti Technical School, **Rapti, Dang, Nepal**

**^3^Departent of Pharmacy, Shree Medical and Technical College, Bharatpur, Nepal**

**Corresponding author**

[*hemrajsharma.hs50@gmail.com](mailto:*hemrajsharma.hs50@gmail.com), ^#^ dcnim2@gmail.com


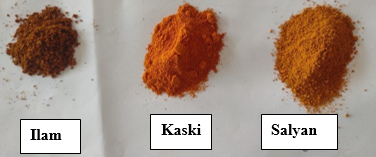


Figure S1: Turmeric powder collected from different places of Nepal

**Description:**

The rhizomes obtained from Ilam, Kaski and Salyan districts of Nepal were shade-dried and homogenized into a powder. Each of the sample was subjected for the extraction and to calculate the yield value of curcumin. The extract with maximum amount of curcumin was used for the method development process.


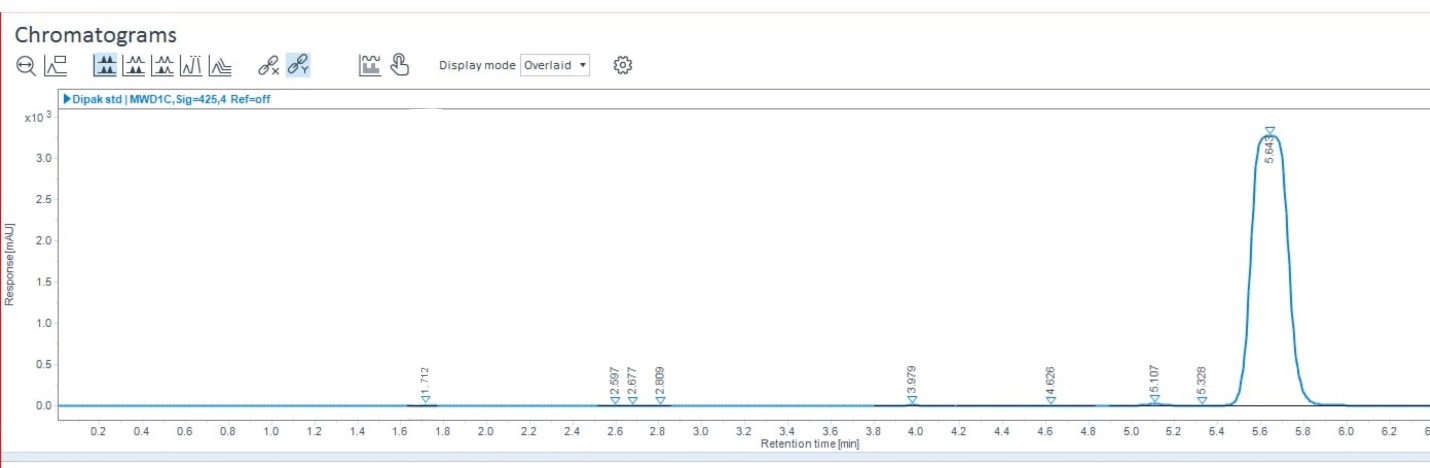

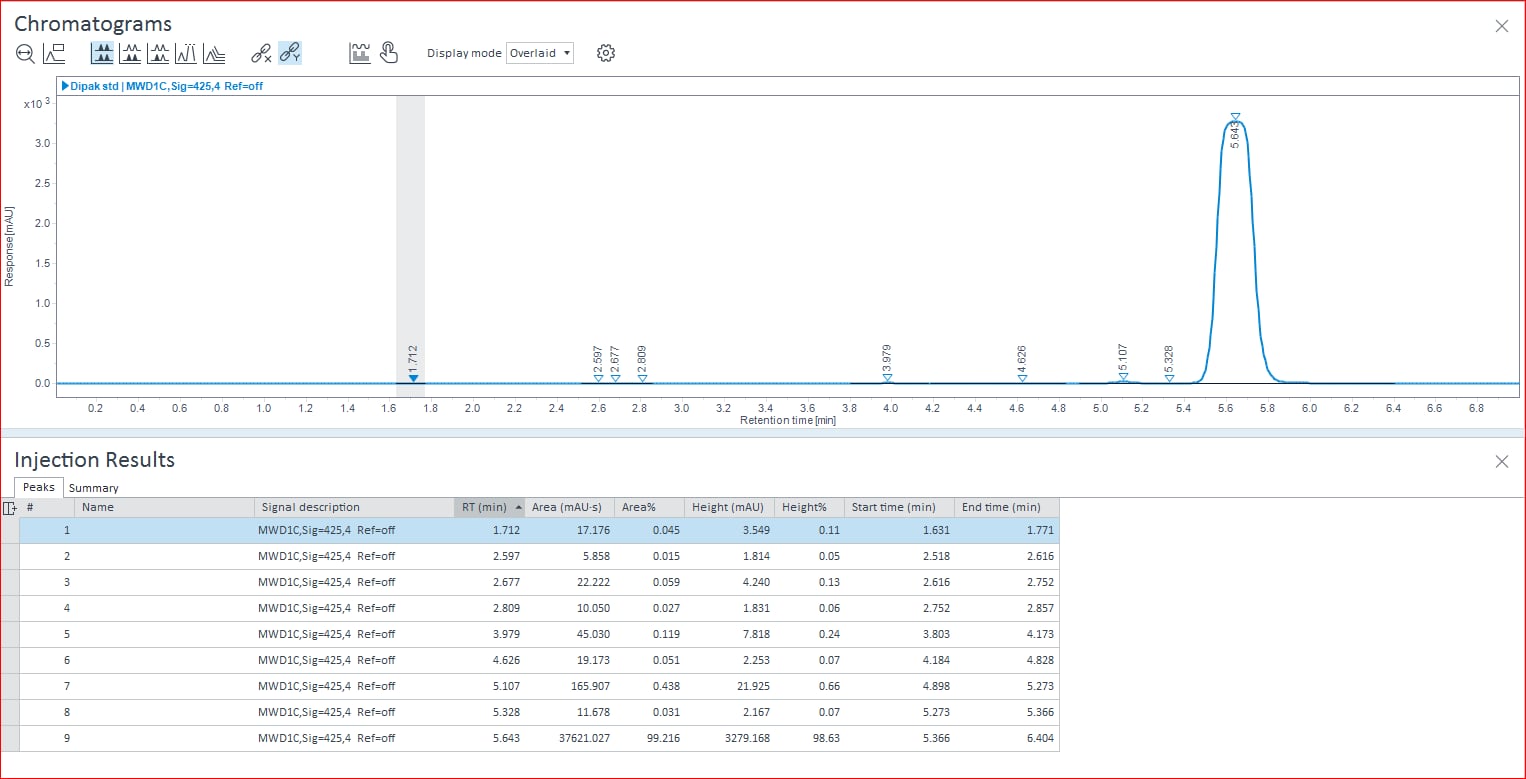


**Figure S2: Reference chromatogram of Curcumin**

**Description:**

In order to analyzed and confirmed the curcumin peak in the sample, a standard curcumin was injecting under identical conditions. In the reference chromatogram, the retention time (t_R_) of curcumin was 5.6; therefore, the peak near 5.6 in the sample was identified as curcumin.

The mobile phase is used was 0.2% acetate buffer and acetonitrile at a ratio of 40:60. The separation of curcumin was achieved in Agilent- Pursuit C18 -150mm ×5mm column with 1.2 ml/min flow rate.

Table S3: Green Analytical Procedure Index (GAPI)

| \| **S.N.** \| **Category** \| **Proposed method** \| **Colour** \| \| --- \| --- \| --- \| --- \| \| **(I) Sample preparation** \| \| \| \| \| 1 \| Collection \| UV \| Green \| \| 2 \| Preservation \| None \| Green \| \| 3 \| Transport \| None \| Green \| \| 4 \| Storage \| None \| Green \| \| 5 \| Type of method: direct or indirect \| Simple procedures \| Yellow \| \| 6 \| Scale of extraction \| Simple extraction using ethanol \| Green \| \| 7 \| Solvents/reagents used \| Green solvents \| Yellow \| \| 8 \| Additional treatments \| None \| Green \| \| 9 \| Reagent and solvent amount \| <10 mL \| Green \| \| 10 \| Health hazard \| None \| Green \| \| 11 \| Safety hazard \| Safe \| Green \| \| **(II) Instrumentation** \| \| \| \| \| 12 \| Energy \| UV consumes ≤0.1 kWh per sample \| Green \| \| 13 \| Occupational hazard (OH) \| None \| Green \| \| 14 \| Waste \| Waste generated by the proposed method was 1–10 mL. \| Yellow \| \| 15 \| Waste treatment \| Low degradation \| Yellow \| |
| --- | --- | --- | --- | --- | --- | --- | --- | --- | --- | --- | --- | --- | --- | --- | --- | --- | --- | --- | --- | --- | --- | --- | --- | --- | --- | --- | --- | --- | --- | --- | --- | --- | --- | --- | --- | --- | --- | --- | --- | --- | --- | --- | --- | --- | --- | --- | --- | --- | --- | --- | --- | --- | --- | --- | --- | --- | --- | --- | --- | --- | --- | --- | --- | --- | --- | --- | --- | --- | --- | --- | --- | --- |
|  |

**Description:**

The GAPI provides a comprehensive evaluation framework that works on NEMI's scope to include additional considerations. It emphasizes the assessment of hazardous solvents by the National Fire Protection Association (NFPA) guidelines, rather than relying solely on EPA standards. GAPI utilizes a Toxic Release Inventory (TRI) list to identify relevant solvents. Notably, its pictogram incorporates three distinct colors green, yellow, and red reflecting varying toxicity levels. This color scheme corresponds to the toxicity of solvents, with red indicating high toxicity, yellow indicating moderate toxicity, and green indicating low or non-toxicity. The application of GAPI in the proposed method is provided in above Table S3, demonstrating that the method satisfies most of the criteria and confirms the proposed method as eco-friendly.

Table S4: Analytical Eco-scale

| **Reagent/instruments** | **Penalty points** |
| --- | --- |
|  | **Proposed spectrophotometric method** |
| Ethanol | 4 |
| Phosphate buffer | 0 |
| Occupational hazards | 0 |
| Waste | 0 |
| Instruments energy | 0 |
| Total penalty points | Σ 100-4= 96 |
| Analytical Eco scale total score | 96 |
| Comment | Excellent green analytical method |

**Description:**

AES generates numerical assessments, with an optimal score capped at 100. The eco-score is calculated based on penalty points assigned for various factors such as reagents used, chemicals used, energy consumption and waste generated. Eco-scale score of 100 indicates the perfect greenness of the method. Score of more than 75, 50-75 and less than 50 indicates excellent, fair, deficient green methods. The eco-scale score of the introduced method was 96, as detailed in above Table S4.

Table S5: Green tool comparison between reference and proposed method

| **Methods** | **GAPI** | **CaFRI** | **CACI** |
| --- | --- | --- | --- |
| Developed method | 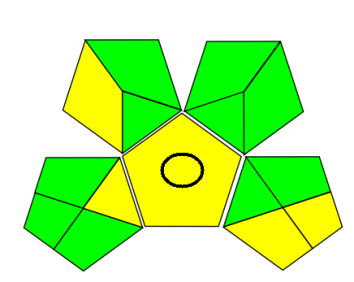 | 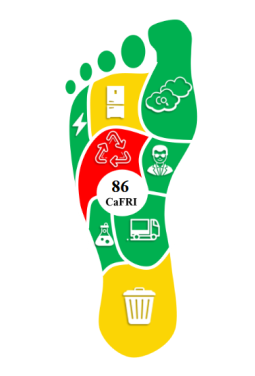 | 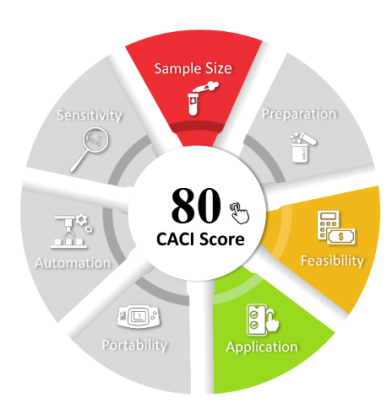 |
| Reference method  (Patelet al. 2022) | **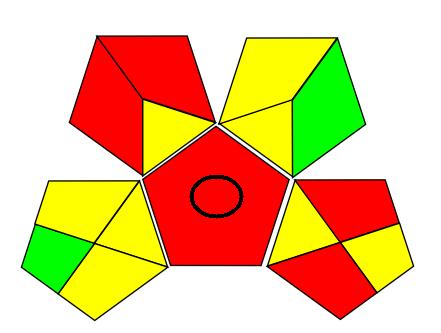** | 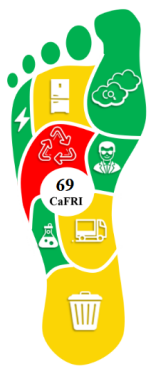 | 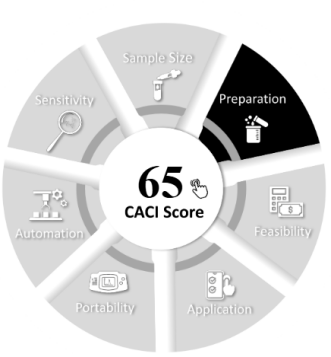 |

**Description:** Patelet al. 2022,(25) developed an analytical method for the estimation of calcium in herbo-mineral formulation (Maxcal-C Tablet) by Atomic Absorption Spectrophotometry and complexometric titration. AAS required significant amounts of energy to operate. This high energy consumption is not aligned with the green chemistry goal of minimizing energy use. Different acids like HCl, HNO_3_, HF, and H_2_O_2_ were utilized for digestion. These reagents can be hazardous, corrosive, and non-environmentally friendly, leading to the generation of toxic waste. The greenness of the reference method was established by GAPI pictogram, which showed the method as less eco-friendly as only few analytical steps were green. The eco friendliness of the reference and developed method was also established, which showed the developed method as eco-friendlier and more sustainable, as shown in above Table S5.
